# Gamabufotalin, a bufadienolide compound from toad venom, suppresses COX-2 expression through targeting IKKβ/NF-κB signaling pathway in lung cancer cells

**DOI:** 10.1186/1476-4598-13-203

**Published:** 2014-08-31

**Authors:** Zhenlong Yu, Wei Guo, Xiaochi Ma, Baojing Zhang, Peipei Dong, Lin Huang, Xiuli Wang, Chao Wang, Xiaokui Huo, Wendan Yu, Canhui Yi, Yao Xiao, Wenjing Yang, Yu Qin, Yuhui Yuan, Songshu Meng, Quentin Liu, Wuguo Deng

**Affiliations:** Institute of Cancer Stem Cell; College of Pharmacy, Dalian Medical University, Lvshun South Road No 9, Dalian, 116044 China; Sun Yat-sen University Cancer Center; State Key Laboratory of Oncology in South China, Collaborative Innovation Canter of Cancer Medicine, Guangzhou, China

**Keywords:** Gamabufotalin, NSCLC, COX-2, NF-κB, p300, IKKβ

## Abstract

**Background:**

Gamabufotalin (CS-6), a major bufadienolide of Chansu, has been used for cancer therapy due to its desirable metabolic stability and less adverse effect. However, the underlying mechanism of CS-6 involved in anti-tumor activity remains poorly understood.

**Methods:**

The biological functions of gamabufotalin (CS-6) were investigated by migration, colony formation and apoptosis assays in NSCLC cells. The nuclear localization and interaction between transcriptional co-activator p300 and NF-κB p50/p65 and their binding to COX-2 promoter were analyzed after treatment with CS-6. Molecular docking study was used to simulate the interaction of CS-6 with IKKβ. The *in vivo* anti-tumor efficacy of CS-6 was also analyzed in xenografts nude mice. Western blot was used to detect the protein expression level.

**Results:**

Gamabufotalin (CS-6) strongly suppressed COX-2 expression by inhibiting the phosphorylation of IKKβ via targeting the ATP-binding site, thereby abrogating NF-κB binding and p300 recruitment to COX-2 promoter. In addition, CS-6 induced apoptosis by activating the cytochrome c and caspase-dependent apoptotic pathway. Moreover, CS-6 markedly down-regulated the protein levels of COX-2 and phosphorylated p65 NF-κB in tumor tissues of the xenograft mice, and inhibited tumor weight and size.

**Conclusions:**

Our study provides pharmacological evidence that CS-6 exhibits potential use in the treatment of COX-2-mediated diseases such as lung cancer.

## Introduction

Lung cancer is a leading cause of human death worldwide, and non-small cell lung cancer (NSCLC) comprises approximately 85% of all lung cancers. Although the diagnostic and therapeutic techniques have been improved in recent decades, poor prognosis of NSCLC still leads to a less than 20% five-year survival rate of the patients [1–3]. Therefore, novel therapy strategies are necessary to improve the survival rate of patients with lung cancer.

NF-κB is a family of important transcriptional factors known to regulate a wide range of biological effects, including proliferation, metastasis and apoptosis, via its downstream target genes [4, 5]. NF-κB is sequestered in the cytoplasm in resting cells by the inhibitory IκB proteins [6]. In response to a variety of stimulation, IκB would be phosphorylated by the inhibitor of κB (IKK) kinase complex and further be degraded through ubiquitination–proteasome pathway [7], thus releasing the active NF-κB dimmers. The free NF-κB then binds to DNA and affects gene expression. Therefore, inhibition of NF-κB activation might be an effective alternative approach to suppress cancer growth.

It has been well documented that COX-2 is a key enzyme involved in cancer development and progression, and plays a central role in the modulation of tumor viability, migration, invasion, and even inflammation [8–10]. COX-2, as the rate-limiting enzymes for the synthesis of prostaglandins from arachidonic acid, is linked to the carcinogenesis of many human cancers, including lung, breast, colon, esophagus, head and neck cancers, and its overexpression is highly related to the poor prognosis of patients [11–16]. Its selective inhibitors could effectively prevent inflammation, proliferation and angiogenesis, and induce apoptosis in human cancer cells. In addition, COX-2 expression is transcriptionally controlled by the binding of multiple transactivators such as NF-κB and coactivators such as p300 and p65 to the corresponding sites of its promoters [17, 18]. Thus, the signals to activate NF-κB have been shown to induce COX-2 expression, implying that the anti-cancer effect of drugs may be concomitant with the downregulation of COX-2 expression.

IKK including IKKα (IKK1) and IKKβ (IKK2), is the convergency point in most signaling pathways activated by many stimuli leading to the inducible phosphorylation and degradation of IκB proteins, thus activated NF-κB. Some studies have clearly demonstrated that although IKKα and IKKβ have a high degree of sequence homology and share similar structural domains, IKKβ has 20–50-fold higher level of kinase activity for IkB than IKKα [19]. Thus, it is essential to identify a small-molecule inhibitor selectively targeting IKKβ, and explore its mechanisms regulating NF-κB activation.

**Figure 1:**
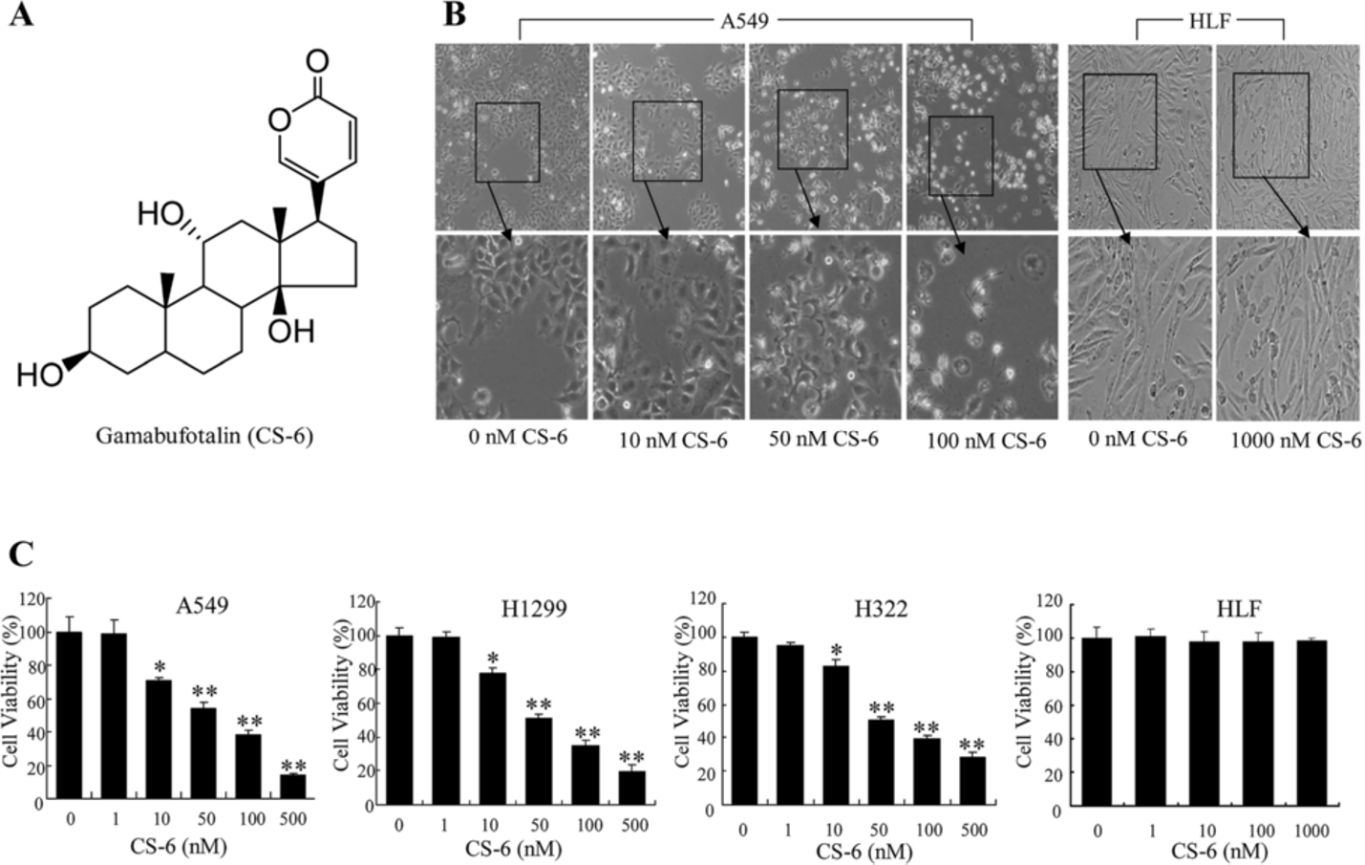
**CS-6 inhibited cell viability and changed morphology. (A)** Chemical structure of CS-6. **(B, C)** Human lung cancer A549, H1299, H322 cells and human embryo lung fibroblast (HLF) cells were treated with CS-6 under normal culture medium at the indicated doses. **(B)** The changes in cell morphology and spreading in A549 cells treated with CS-6 for 48 h were observed and cells were photographed using a microscope fitted with digital camera. **(C)** At 48 hours after treatment, the cell viability was determined by a MTT assay. The data are presented as mean ± SD of three tests. (*P < 0.05,**P < 0.01, significant differences between CS-6treatment groups and DMSO vehicle control groups).

In recent years, increasing attentions have been paid on some natural products that existing in traditional Chinese medicines due to their great potential in various cancer therapies [20]. Toad venom, called “Chansu” (CS) in China, from the postauricular glands and skin of Bufo bufo gargarizans Cantor [21, 22] have been widely and successfully used for centuries alone or in combination with other herbal ingredients, as an anodyne, cardiotonic, antimicrobial, local anesthetic, anti-inflammatory and antineoplastic agent [23]. Gamabufotalin (CS-6, Figure 1A), a major derivative of bufadienolides, had higher content of 1.75%-5%, and significant anti-tumor activity [24]. And our previous work (data not shown) further supports its stable metabolic property and less adverse effects when compared with other bufadienolides. However, as a bioactive molecular, the detailed anti-cancer mechanism of CS-6 had not yet been characterized.

In this study, we investigated the mechanisms of CS-6 for COX-2 suppression *in vivo* and *in vitro*, and revealed that CS-6 could target IKKβ to inactive NF-κB signaling pathway and further down-regulate COX-2 expression. Our findings therefore suggested that CS-6 would serve as a potential candidate targeting IKKβ to suppress COX-2 expression for anticancer treatment.

## Materials and methods

### Chemicals and reagents

Gamabufotalin (CS-6) was isolated from ChanSu by Dr. Xiaochi Ma (Dalian Medical University, Liaoning, China), which was secreted from the postauricular and skin glands of Bufo bufo gargarizans Cantor. The crude materials of ChanSu were purchased from Qingdao, Shandong Province of China. And a voucher specimen had been deposited at School of Pharmacy, Dalian Medical University. A preparative high-speed counter-current chromatography (HSCCC) method for isolation and purification of bufadienolides from ChanSu was developed by using a stepwise elution with two-phase solvent system composed n-hexane-ethyl acetate- methanol–water at the ratios of 4:6:2:4 (v/v), 4:6:2.5:4 (v/v) and 4:6:3.2:4 (v/v). A total of 38 mg of CS-6 was obtained in one-step separation from 1.1 g of the crude extract with purity of 98.7%. Its chemical structure was identified on the basis of ^1^H-NMR and ^13^C-NMR technology. All organic solvents for HSCCC separation and preparation of crude extract were the analytical grade (Beijing Chemical Factory, China). For our experiments, the solution of CS-6 (1 μM) in dimethyl sulphoxide (DMSO) was prepared and kept at -20°C, as the stock solution. CS-6 was diluted in culture medium to obtain the desired concentration, which was stable in the dilution with DMSO concentration less than 0.1%. The Phosphate Buffered Saline (PBS), protease inhibitor cocktail and 5-diphenyltetrazolium bromide (MTT) were purchased from Sigma Chemical Co (St. Louis, MO).

### Antibodies and other materials

The primary antibodies for COX-2, IKKα, IKKβ, p-IKKα/β, IκB-α, p-IκB-α, cleaved caspase-9, NF-κB p65 and p-p65, β-actin and all the secondary antibodies were obtained from Cell Signaling Technology (Cell Signaling Technology, Inc, USA). The primary antibodies for GAPDH, p300, NF-κB p50, and cytochrome c were obtained from Santa Cruz Biotechnology (Santa Cruz, CA, USA). Trypsin, Dulbecco’s Modified Eagle’s Medium (DMEM), RPMI 1640 and fetal bovine serum (FBS) were obtained from HyClone Laboratories (HyClone Laboratories Inc.). All other chemicals were purchased from Sigma Chemical Co. (St. Louis, MO) unless otherwise specified.

### Cell culture

Human NSCLC H1299, A549, H322 and H460 cell lines and human embryonic lung fibroblasts HLF cell line were obtained from ATCC (Manassas, VA). Cells were maintained in either DMEM medium or RPMI 1640 medium supplemented with 10% fetal bovine serum. All cell cultures were maintained at 37°C in a humidified atmosphere containing 5% CO_2_.

### Cell viability assay

Cell viability was determined by a MTT assay (Roche Diagnosis, Indianapolis, IN). Briefly, lung cancer cell lines were seeded at 6 × 10^3^ cells/well in 96-well plates. Cells were allowed to adhere for overnight, and then the cells were changed to fresh medium containing various concentrations of CS-6 (0, 10, 50 and 100 nM) dissolved in DMSO (final concentration, 0.1%). After 48 h incubation, the growth of cells was measured. The effect on cell viability was assessed as the percent cell viability compared with untreated control group, which were arbitrarily assigned 100% viability. The CS-6 concentration required to cause 50% cell growth inhibition (IC_50_) was determined by interpolation from dose–response curves. All experiments were performed in triplicate.

### *In vitro*migration assay

Scratch assay (wound healing assay) was performed to detect cell migration. The cells were grown to full confluence in six-well plates and wounded with a sterile 100 ul pipette tip after 6 h of serum starvation and then washed with starvation medium to remove detached cells from the plates. Cells were treated with indicated doses of CS-6 in full medium and kept in a CO_2_ incubator. After 48 h, medium was replaced with phosphate buffered saline (PBS) buffer, the wound gap was observed, and cells were photographed using a Leica DM 14000B microscope fitted with digital camera.

### Colony formation assay

To analyze the cell sensitivity to CS-6, we used a colony formation assay *in vitro*. Briefly, A549 cells (0.8 × 10^3^ per well) were seeded in six-well plate containing 2 ml growth medium with 10% FBS and cultured for 24 h. Then, removed the medium, and cells were exposed to various concentrations of CS-6 (0, 10, 50 and 100 nM). After 24 h, cells were washed with PBS and supplemented fresh medium containing 10% FBS. The cultures were maintained in a 37°C, 5% CO_2_ incubator for 14 days, allowing viable cells to grow into macroscopic colonies. Removed the medium, and the colonies were counted after staining with 0.1% crystal violet.

### Apoptosis assay

Apoptosis was measured by fluorescence-activated cell sorter (FACS) using the Annexin V- FITC Apoptosis Detection Kit (Nanjing KeyGEN Biotech. CO., LTD.). In brief, cells plated in 6-well plates were treated with CS-6. After treatment of 12 h, cells were collected and washed once with cold PBS, and subsequently stained simultaneously with FITC-labeled annexin V and PI. Stained cells were analyzed using FACS Accuri C6 (Genetimes Technology Inc.).

### Western blot analysis

Cell lysate proteins were separated by electrophoresis on a 7.5-12% sodium dodecyl sulfate-polyacrylamide minigels (SDS-PAGE) and then electrophoretically transferred to a PVDF membrane. Western blots were probed with the specific antibodies. The protein bands were detected by enhanced chemiluminescence. Similar experiments were performed at least three times. The total protein concentration was determined using a BCA protein assay kit.

### Confocal immunofluorescence

Immunofluorescence staining was done in cells cultured in chamber slides. After CS-6 treatment, the cells were washed in phosphate-buffered saline (PBS) and fixed for 10 min at room temperature (RT) with 4% paraformaldehyde. The samples were permeabilized with 0.2% TritonX-100 for 5 min. And then blocked with 10% bovine serum albumin (BSA) in PBS for 30 min. Antibodies against Cytochrome c, p65, p50, and p300 in the 1% blocking solution were added to the sample and incubated for overnight at 4°C. Following three 10-min washes with PBS, fluorescein isothiocyanate- and rhodamine-conjugated secondary antibodies were added in 1% blocking solutions and incubated for 1 hr. Subsequently, the stained samples were mounted with 4′, 6-diamidino-2-phenylindole (DAPI)-containing Vectashield solution (Vector Laboratories Inc.) to counterstain cell nuclei. After five additional 5-min washes, samples were examined with a Leica DM 14000B confocal microscope.

### DNA-protein binding by streptavidin-agarose pulldown assay

Binding of p300 or p65, p50 NF-κB to COX-2 core promoter probes were determined by a streptavidin-agarose pulldown assay. A biotin-labeled double-stranded probe corresponding to COX-2 promoter sequence was synthesized. The binding assay was performed by mixing 400 μg of nuclear extract proteins, 4 μg of the biotinylated DNA probe and 40 μl of 4% streptavidin-conjugated agarose beads at room temperature for 5 h in a rotating shaker. Beads were pelleted by centrifugation to pull down the DNA-protein complex. DNA-bound p300 or p65, p50 NF-κB protein was dissociated by boiled in 30 μl of 2X Laemmli sample buffer and analyzed by Western blotting.

### Reverse transcription-polymerase chain reaction (RT-PCR)

Z138 cells were treated with different concentrations of CS-6 (0, 10, 50, and 100 nM) for 48 h and then harvested. Total cellular RNA was extracted using the RNAiso Plus reagent, according to the manufacturer’s protocol (TaKaRa Biotechnology, Dalian, China). Total RNA (1.5 ug) was reverse-transcribed using the PrimeScriptTM RT-PCR Kit (TaKaRa). PCR analysis was performed on aliquots of the cDNA preparations to detect gene expression. PCR was programmed as follows: 5 min at 95°C followed by 35 cycles (30 for GAPDH), 30 s at 94°C, 30 s at 60°C, and 1 min at 72°C. RT-PCR products were analyzed via 1.0% agarose gel electrophoresis and stained with Gold View for visualization using ultraviolet light.

### Quantitative real-time reverse transcription polymerase chain reaction (RT-qPCR)

Total RNA was extracted from A549 cells after treatment with CS-6 for 48 h, using TRIzol reagent according to the kit protocol (TaKaRa Bio, Dalian, China). cDNA was reverse-transcribed using the PrimeScript RT Reagent Kit (TaKaRa Bio, Dalian, China) according to the manufacturer’s instructions. The Q-PCR reaction was performed following the kit protocol (TaKaRa Bio, Dalian, China), and amplification was performed using the Mx3005P Real-Time PCR System (Agilent, CA, USA). The relative mRNA expression of each gene was normalized to GAPDH RNA levels and analyzed using the 2^-ΔΔCT^ method. The primers were synthesized by invitrogen (Shanghai, China). The primers for COX-2 were: 5′-TCACAGGCTTCCATTGAC CAG-3 and 5′-CCGAGGCTTTTCTACCAGA-3′; the primers for β-actin were: 5′-GGCACCCAGCACAATGAA-3′ and 5′-TAGAAGCATTTGCGGTGG -3′.

### Chromatin immunoprecipitation

The chromatin immunoprecipitation (ChIP) assay was performed as previously described. In brief, about 80% confluent cells were used for the experiments after exposed to CS-6 for 48 h. 1% paraformaldehyde was added to the cell-culture medium to cross-link, and after incubation for 10 min at 37°C with gently shaking, the cells were washed twice in cold phosphate-buffered saline, scraped, and lysed in lysis buffer (1% SDS, 10 mM Tris–HCl, pH 8.0, with 1 mM phenylmethylsulfonyl fluoride, pepstatin A, and aprotinin) for 10 min at 4°C. The lysates were sonicated five times for 15 s each time, and the debris was removed by centrifugation. A 20-μl aliquot was removed to serve as an input sample. The remaining of the lysate were diluted 10-fold with a dilution buffer (0.01% SDS, 1% Triton X-100, 1 mM EDTA, 10 mM Tris–HCl, pH 8.0, and 150 mM NaCl) followed by incubation with antibodies against specific transactivators or a nonimmune rabbit IgG control overnight at 4°C. Immunoprecipitated complexes were collected using protein A/G plus agarose beads. The precipitates were extensively washed and incubated in an elution buffer (1% SDS and 0.1MNaHCO3) at room temperature for 20 min. Cross-linking of protein–DNA complexes was reversed at 65°C for 5 h, followed by treatment with 100 ug/mL proteinase K for 2 h at 37°C. DNA was extracted three times with phenol/chloroform and precipitated with ethanol. The pellets were resuspended in TE buffer and subjected to PCR amplification using specific COX-2 promoter primers (Forward primer: ACGTGACTTCCTCGACCCTC, and Reverse primer: AAGACTGAAAA CCAAGCCCA). The resulting product of 478 bp for COX-2 in length was separated by 1.0% agarose gel electrophoresis.

### Molecular modeling

The molecular docking studies were performed to explore the potential binding mode between CS-6 and IKKβ protein complex. CS-6 was optimized using the semi-empirical PM3 method with the Polak-Ribie’re conjugate gradient algorithm with an RMS gradient of 0.01 kcal mol^-1^ Å^-1^ as convergence criterion. The optimized structure of CS-6 was docked into the active site of IKKβ with ligand K-252A (PDB Code: 4KIK). The crystallographic ligand was extracted from the active site, and the residues within a 6.5 A° radius around IKKβ molecule were defined as the active pocket. The Surflex-Dock program was used for the docking calculations with default parameters. MOLCAD surfaces were generated for visualizing the binding mode of the docked protein–ligand complexes.

### Animal studies

All animals were maintained, and animal experiments were done in SPF Laboratory Animal Center at Dalian medical university. The animals used in this study were female nu/nu mice (4–6 weeks old). To evaluate the therapeutic efficacy of CS-6 in a human A549 orthotopic lung cancer mouse model, A549 cells (2 × 10^6^ in 100 μL PBS) were injected subcutaneously near the axillary fossa of the nude mice using a 27-gauge needle. The tumor cell–inoculated mice were randomly divided into three treatment groups that each contained five mice. Two weeks later, when the tumor diameters reached 3 mm × 4 mm, group A was treated with PBS; group B with 100 ug/20 g CS-6; group C with 100 ug/20 g CS-6 by intraperitoneal injection every day. Tumors were measured with a caliper every 2 days, and the tumor volume was calculated using the formula V = 1/2 (width^2^ × length). Body weights were also recorded. On day 30 after tumor cell inoculation, all experimental mice were terminated with ether anesthesia and the total weight of the tumors in each mouse was measured. To determine COX-2 or p65 NF-κB expression, the tumor tissues were harvested and freshly fixed with 10% neutral formalin and desiccated and embedded in paraffin. 4 μm sections were stained with hematoxylin and eosin, COX-2 antibody (1:80) and p-p65 NF-κB (1:150) antibody, and examined under a light microscope. The images were examined under a Leica DM 4000B fluorescence microscope equipped with a digital camera.

All animal maintenance and procedures were carried out in strict accordance with the recommendations established by the Animal Care and Ethics Committee of Dalian Medical University as well as the guidelines by the U.S. National Institutes of Health Guide for the Care and Use of Laboratory Animals. The protocol was approved by the Animal Care and Ethics Committee of Dalian Medical University. In animal study, all efforts were made to minimize suffering of mice. All mice were humanely sacrificed by ether anesthesia inhalation before death.

### Statistical analysis

All experiments were repeated three times. Data are represented as mean ± standard deviation (SD). Analysis of variance and Student’s *t*-test were used to compare the values of the test and control samples *in vitro* and *in vivo*. P < 0.05 was considered to be a statistically significant difference. SPSS 17.0 software was used for all statistical analysis.

## Results

### CS-6 inhibited NSCLC cell proliferation and changed cell morphology

Cell viability plays an essential role in carcinogenesis, and its inhibition is crucial to treat cancer [25]. Firstly, we quantitatively examined the effect of CS-6 (Figure 1A) on cell morphology and cell proliferation of several human lung cancer cell lines by MTT assay. As shown in Figure 1B, CS-6 markedly reduced cell-to-cell contact and had lower spreading with fewer formation of filopodia compared with the DMSO vehicle control groups. Interestingly, treatment with CS-6 resulted in the dose-dependent growth inhibition of NSCLC cells, but has no cytotoxicity in human normal lung cell line (HLF cells) at the same dose (Figure 1C).

### CS-6 suppressed colony formation and migration of NSCLC cells

**Figure 2:**
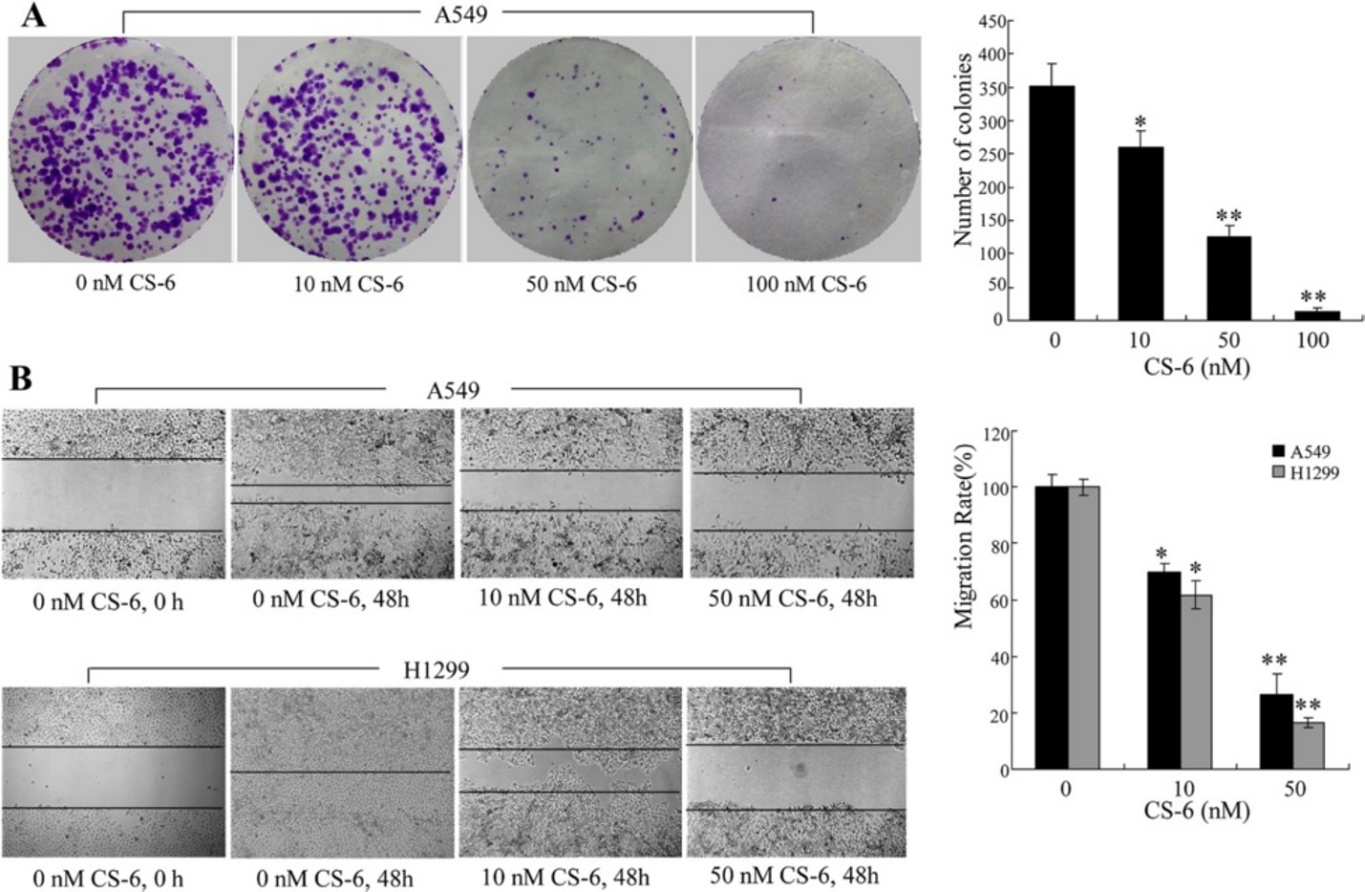
**CS-6 suppressed cell colony formation and migration. (A-B)** Human A549 and H1299 cells were treated with CS-6 at the indicated doses for appropriate time. **(A)** The tumor cell A549-induced colony formation was also analyzed, and the colony formation rate was calculated. **(B)** Cell migration was analyzed by a wound-healing assay. A549 and H1299 cells were seeded in 6-well plates and grown to full confluence. Cell migration was measured as described in Section 2, and the migration rate was calculated. The data are presented as the mean ± SD of three separate experiments. (*P < 0.05,**P < 0.01, significant differences between CS-6treatment groups and DMSO vehicle control groups).

Clonogenic cell survival assay was employed to evaluate the influence of CS-6 on the clonogenic capacity of A549 cells. Consistent with cell proliferation inhibition, CS-6 also significantly inhibited colony formation and resulted in a remarkable decrease at colony formation ratio (Figure 2A). Wound-healing assay further revealed the inhibition effect of CS-6 on tumor cell mobility in A549 and H1299 cells. As shown in Figure 2B, the part of gap or wounding space between cell layers after making a scratch was occupied almost (in A549 cells) or completely (in H1299 cells) by the migrating cells after 48 h in the control group. By contrast, the CS-6 treated cells failed to occupy the scraped space through migration due to their impaired migration capability. Quantitative analysis revealed that the inhibition of migration was in a dose-dependent manner. These results suggest that CS-6 has the perfect properties in suppressing cell colony formation and migration for NSCLC cells.

### CS-6 induced apoptosis via modulating cytochrome c and caspase signaling

Apoptosis is a cell suicide mechanism that enables organisms to control cell numbers and eliminate malignant cells that threaten survival [26]. Induction of apoptosis in target cells is a key mechanism for anti-cancer therapy [27]. We next determined whether the enhancement of cell growth inhibition induced by CS-6 is associated with the increase of apoptosis in lung cancer cells. We conducted a time-course study for the effect of CS-6 on apoptosis using two effective concentrations. The results showed that CS-6 treatment resulted in a significant dose-dependent induction of apoptosis in A549 cells. We also found that the induction of apoptotic cells by CS-6 had significant difference between 12 h and 24 h or 48 h, but had no difference between 24 h and 48 h.

**Figure 3:**
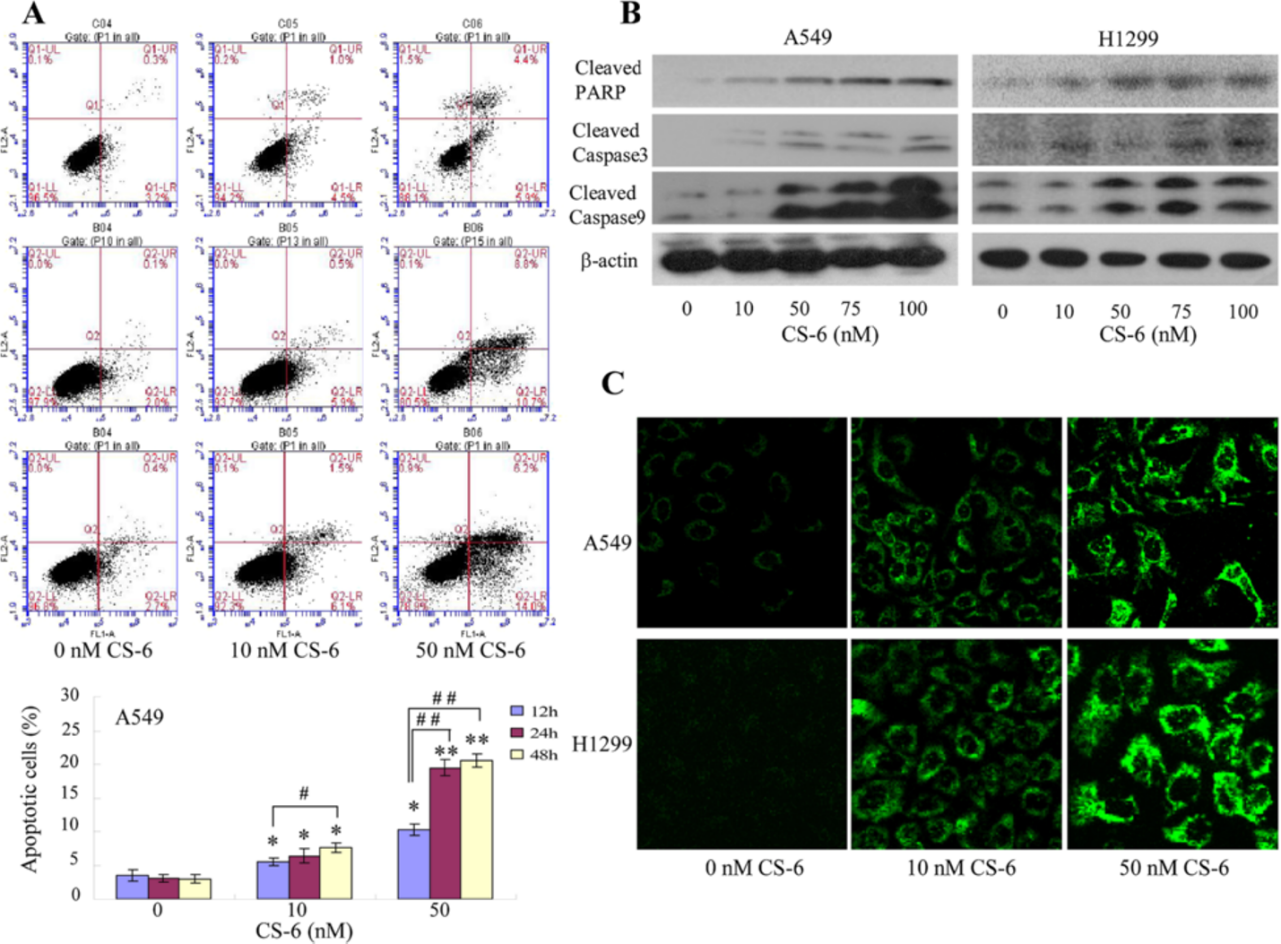
**CS-6 induced apoptosis by modulating cytochrome c/caspase signaling.** Human A549 cells were treated with CS-6 at the indicated doses. Treatment with CS-6 in a time-course manner, the apoptosis was determined by a FACS analysis **(A)**, and the levels of the cleaved caspase-3/9, cleaved PARP proteins in A549 and H1299 cells was analyzed by Western blot **(B)**. The release of Cytc in A549 and H1299 cells was determined by immunofluorescence imaging analysis to monitor Cytc release from the mitochondrial intermembrane space into the cytosol **(C)**. The apoptosis is represented by relative percentages of apoptotic cells versus that in DMSO-treated cells. (*P < 0.05,**P < 0.01, significant differences between CS-6 treatment groups and DMSO vehicle control groups; ^#^P < 0.05, ^##^P < 0.01, significant differences between 48 h treatment group and 12 h treatment group).

To make sure the effect of CS-6 on cell apoptosis, then we detected the expression of three key pro-apoptotic proteins (PARP, caspase-3, caspase-9) in both A549 and H1299 cells by Western blot analysis. CS-6 could markedly increase the expression levels of the cleaved caspase-3, caspase-9 and PARP proteins as compared with the control group (Figure 3B). Additionally, some evidences had pointed out that the release of cytochrome c (cyt c) from mitochondria into cytosol is a critical step in the activation of apoptosis [28]. Many apoptotic stimuli induce cyt c release from the mitochondrial intermembrane space into the cytosol, thereby inducing apoptosis. We next monitored the changes in the subcellular localization of cyt c in A549 and H1299 cells to determine whether CS-6 could induce cyt c release by employing immunofluorescence imaging (IF) analysis. As shown in Figure 3C, treatment with CS-6 (10 nM or 50 nM) markedly triggered the release of cyt c from mitochondria to cytosol. These results demonstrated that CS-6 could induce NSCLC cell apoptosis through triggering cyt c release from mitochondria and facilitating the downstream apoptosome assembly and caspase activation in the cytosol.

### CS-6 suppressed COX-2 expression in NSCLC cells

**Figure 4:**
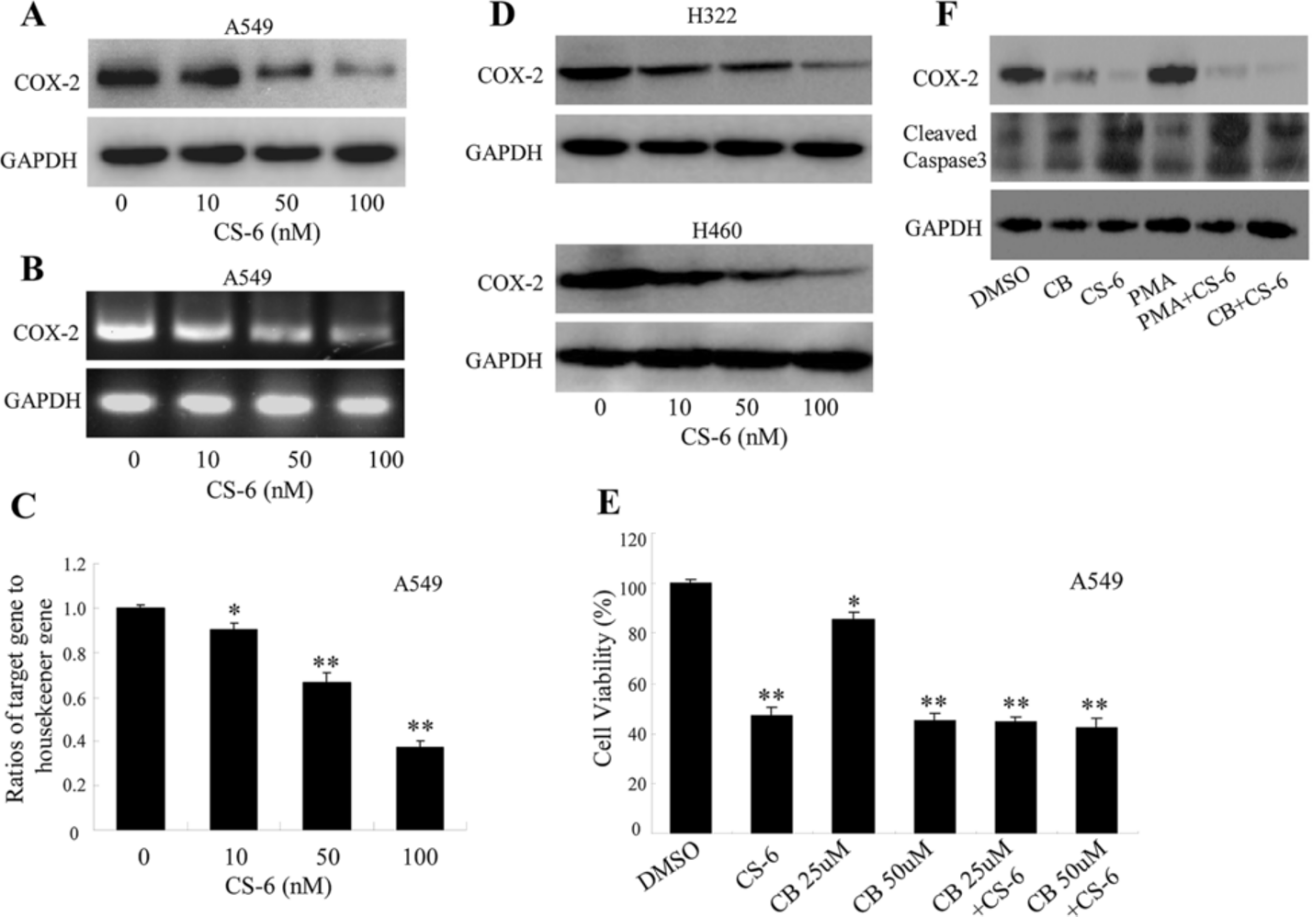
**CS-6 suppressed COX-2 expression. (A–C)**, Human A549, H322, H460 cells were treated with CS-6 at the indicated doses. At 48 h after treatment, expression levels of COX-2 protein and gene were analyzed by Western blotting **(A)**, RT-PCR **(B)** and RT-qPCR **(C)** in A549 cells, respectively. **(D)** At 48 h after treatment, the COX-2 protein levels were analyzed by Western blotting in H322 and H460 cells. GAPDH was used as controls for sample loading. **(E)** A549 cells were pretreated with the COX-2-selective inhibitor celecoxib (CB, 25 uM and 50 uM) for 8 h and then treated with CS-6 (50 nM). At 48 h after treatment, cell viability was determined by MTT analysis. The percent cell viability was calculated relative to the cells treated with the DMSO vehicle control. The data are presented as the mean ± S.D. of three separate experiments. (*P < 0.05,**P < 0.01, significant differences between CS-6treatment groups and DMSO vehicle control groups). **(F)** A549 cells were pretreated with the COX-2 inhibitor celecoxib (CB, 50 uM) and inducer pyromellitic acid (PMA, 200 nM) for 8 h and then treated with CS-6 (50 nM). The protein levels of COX-2, the expression of cleaved caepase-3 were analyzed by Western blot.

COX-2 expression has been demonstrated to induce cell proliferation, migration, and angiogenesis in cancer cells. To determine whether CS-6 influenced COX-2 signaling in lung cancer cells, expression level of COX-2 gene and protein in the CS-6 treated cancer cells was detected by RT-PCR, RT-qPCR and Western blot, respectively. As shown in Figures 4A,B and C, the CS-6 effectively inhibited COX-2 expression at both protein and mRNA levels in A549 cells in a dose-dependent manner. To determine whether CS-6 also inhibited COX-2 expression in other NSCLC cells, we treated H322 and H460 cells with CS-6 at different doses, and found that CS-6 also considerably suppressed COX-2 protein expression in both H322 and H460 cells.To further confirm the role of CS-6 in regulating the COX-2 signaling in lung cancer cells, A549 cells were treated with CS-6 (50 nM) after COX-2-selective inhibitor celecoxib (CB, 25 uM and 50 uM) pretreatment at 8 h. After continuous incubation of 48 h, the cell proliferation was analyzed by a MTT assay. As shown in Figure 4E, treatment of cells with different dosage of CB alone has different inhibitory effect on cell viability, however, the combined treatment with CS-6 (50 nM) and CB did not markedly affect the cell viability compared with CS-6 treatment alone. These results suggested that CS-6 might also partially inhibit the activation of the COX-2 signaling, thereby affecting cell viability.To see whether there was a correlation between the COX-2 inhibition and apoptosis induction mediated by CS-6 in NSCLC cells, we co-treated A549 cells with CS-6 in combination with a COX-2 inhibitor (CB) or a COX-2 inducer (PMA), and then analyzed the expression of COX-2 and the apoptosis-related protein (cleaved caspase-3) by Western blot. The results showed that treatment of A549 cells with the COX-2 inhibitor (CB) or inducer (PMA) didn’t markedly affect the expression levels of the cleaved caspase-3, suggesting that there was no obvious correlation between the CS-6-mediated COX-2 inhibition and apoptosis induction in NSCLC cells (Figure 4F).

### CS-6 inhibited NF-κB and p300 translocation and binging to COX-2 promoter

**Figure 5:**
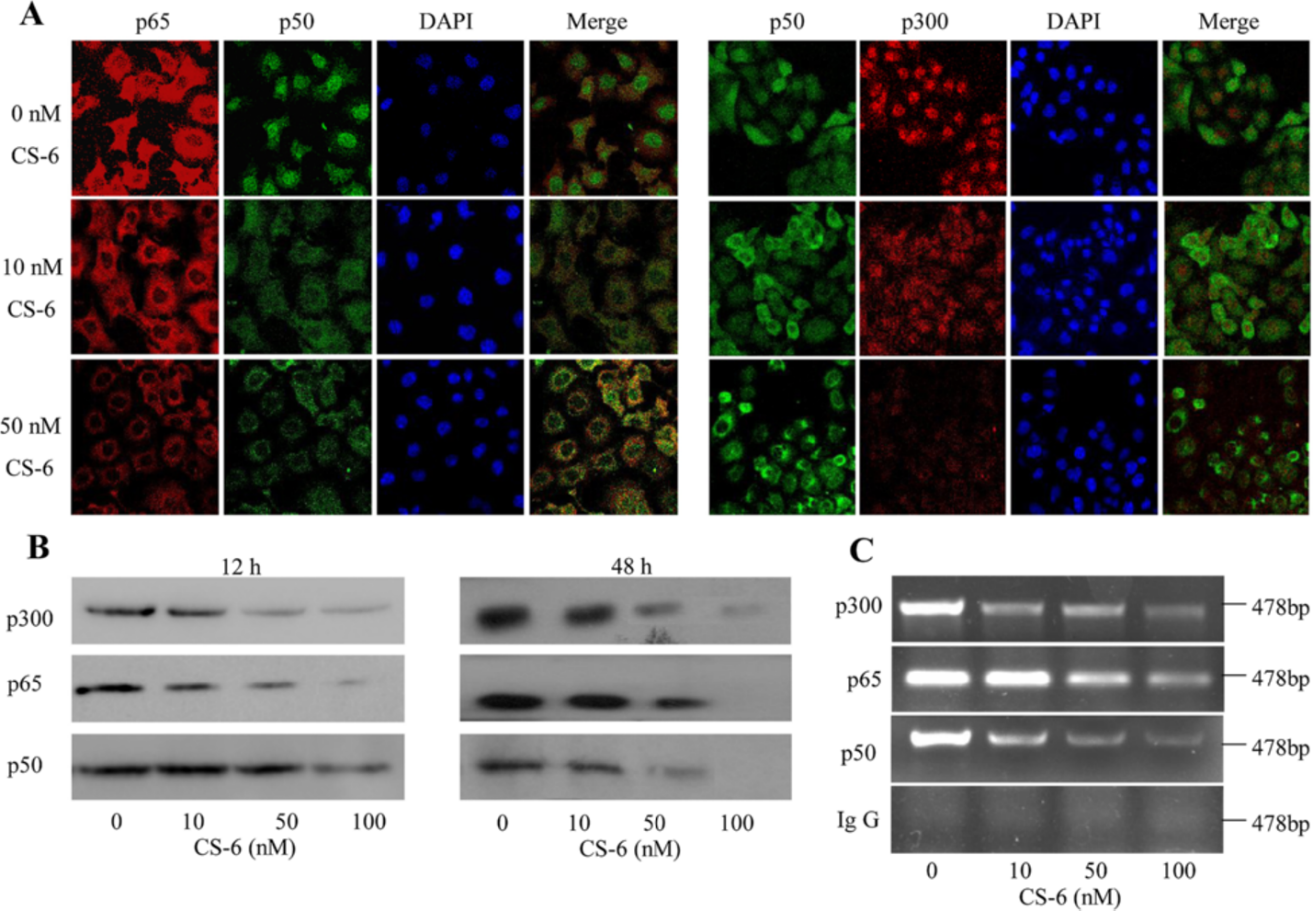
**CS-6 inhibited NF-κB and p300 translocation and their binging to COX-2 promoter. (A-C)** Human A549 cells were treated with CS-6 at the indicated doses. **(A)** At 12 h after treatment, the subcellular localization of p50, p65, and p300 and the colocalization of p65 with p50 or p300 were examined by confocal microscopy analysis with a confocal microscope. More than 100 cells were inspected per experiment, and cells with typical morphology were presented. **(B)** At 12 and 48 h after treatment, the binding of p300, p65 and p50 to COX-2 promoter probe was analyzed by a streptavidin-agarose pulldown assay. **(C)** At 48 h after treatment, chromatin in the treated cells was immunoprecipitated with antibodies to p50, p65, and p300, and the COX-2 promoter region in the precipitated chromatin was amplified by PCR.

Previous reports suggested that NF-κB is an important transcription factor that regulates the expression of COX-2 and inflammatory cytokines [4, 29], the region of COX-2 gene promoter contains a binding sequence for NF-κB [30, 31], and transcriptional coactivator p300 can bind to promoter-bound transactivators such as NF-κB to regulate COX-2 gene expression in cancer cells. We next performed immunofluorescence assay to confirm the nuclear localization and interaction between p300 and NF-κB in A549 cells. Constitutive translocation of NF-κB p50/p65 and coactivator p300 to the cell nucleus and the colocalization of p50 with p65 and p300 were detected in A549 cells. Treatment with CS-6 markedly inhibited translocation of the NF-κB p65/p50 proteins from cell cytoplasm to nucleus and caused p300 into the cytoplasm by comparison with the DMSO control (Figure 5A). The results indicate that the inhibition of A549 cell proliferation by CS-6 might be mediated by inhibiting NF-κB and p300 translocation form cell nuclei to cytoplasm to further inhibit COX-2 expression.We further evaluated the effect of CS-6 on the binding activities of NF-κB and p300 on COX-2 promoter by streptavdin-agarose pulldown (Figure 5B) and ChIP assay (Figure 5C). The results showed that treatment of cells with CS-6 for 12 h or 48 h markedly inhibited the binding of NF-κB p50 and p65 subunits to the COX-2 promoter DNA probe (Figure 5B) or to the COX-2 promoter in chromatin structure (Figure 5C) in a dose-dependent manner as compared with the control treatment. Moreover, we found that p300 bound to the NF-κB responsive element region of the COX-2 promoter as a co-activator, while CS-6 treatment also dose-dependently inhibited the binding of p300 to the COX-2 promoter at 12 h or 48 h treatment. These results suggest that the inhibition of COX-2 expression by CS-6 might be mediated by modulating the NF-κB/p300 signaling pathway in lung cancer cells.

### CS-6 inhibited IKKβ activity by targeting the ATP binding site

**Figure 6:**
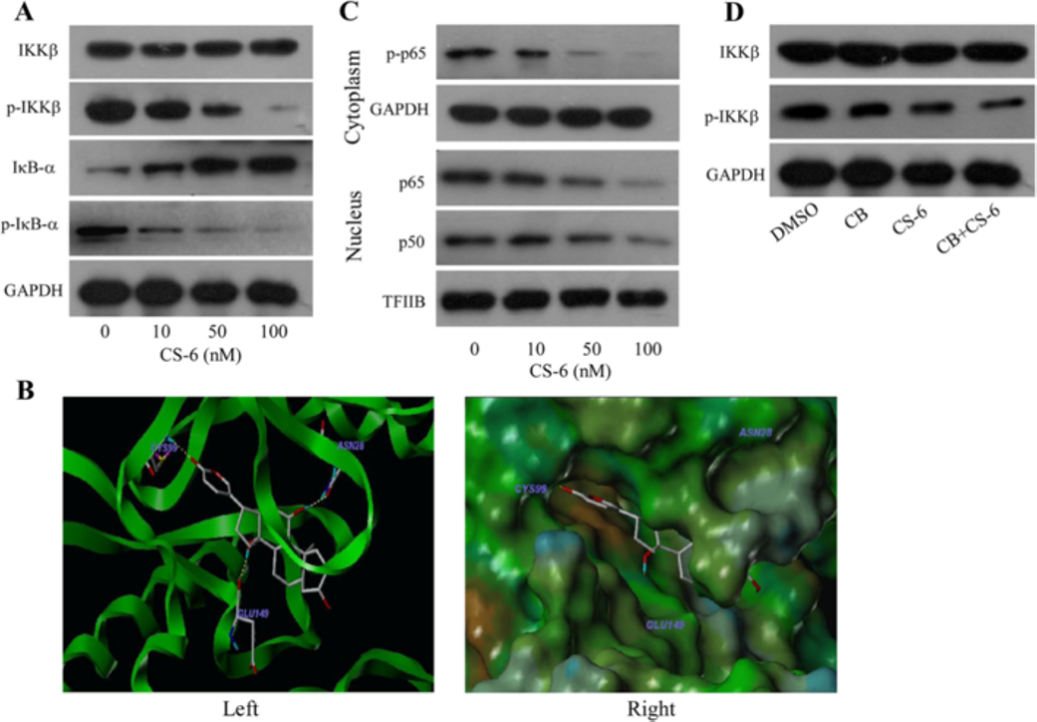
**CS-6 inhibited the phosphorylation and activation of IKKβ. (A)** Human A549 cells were treated with CS-6 at the indicated doses. At 48 h after treatment, the IKKβ, p-IKKβ, IκBα and p-IκBα proteins were analyzed by Western blotting. **(B)** The best ranked pose of CS6 in the ATP binding site of IKKβ generated with docking. (Left) Interactions of CS6 and IKKβ are delineated by ribbon structure, Hydrogen bonds are displayed as yellow dashed lines, and the participating amino acid residues are marked. (Right) MOLCAD representation the molecular lipophilic potential surface upon the bioactive pose of CS6 in the ATP binding site of IKKβ. The blue denotes the hydrophilic, brown for the lipophilic and green corresponds to the neutral moiety. **(C)** Cytoplasmic and nuclear extracts were prepared for the Western blot analysis of p-p65, p65 and p50. GAPDH and TFIIB were used as controls for sample loading. **(D)** A549 cells were treated with CS-6 (50 nM) after pretreatment with CB (50 nM). The IKKβ, and p-IKKβ proteins were analyzed by Western blotting.

Since IκB kinase (IKK) complex is required for NF-κB activation, and its one member, IKKβ, is a major upstream kinase of IκB-α canonical NF-κB signaling pathway [19], we then investigated whether CS-6 exert some influence on IKK activity with an aim to better understand the related molecular events.. As shown in Figure 6A, pretreatment with CS-6 not only significantly suppressed the phosphorylation of p-IKKβ in A549 cells without affecting their overall IKKβ expression, but also decreased the expression level of p-IκB-α. Thereafter, we hypothesized that CS-6 might bind to IKKβ and subsequently inhibited its kinase activity. To test this hypothesis, computer molecular modeling assay was conducted to simulate the interactions between CS-6 and IKKβ. Molecular docking studies predicted that CS-6 could bind to ATP binding site of IKKβ. Specifically, as shown in Figure 6B (Left), CS-6 formed three hydrogen bonds with the ATP binding pocket of the IKKβ kinase domain. The CO motif at the lactonic ring of CS-6 forms a hydrogen bond with the backbone NH of Cys99. The OH group at the C-30 position forms an additional hydrogen bond to the carbonyl oxygen of Glu149. Moreover, the OH group at the C-14 position accepts a hydrogen bond with the CO of Asn28. The result of MOLCAD surface modeling indicated that the lacton ring of CS-6 extends into the deep hydrophobic cavity of the ATP-binding pocket (Figure 6B, Right). As expected, both p-p65 expression level in cytoplasmic and p65, p50 expression in nuclear decreased significantly after CS-6 treatment (Figure 6C). All of these results supported that IKKβ was a target of CS-6 in the NF-κB signaling pathway to suppressed COX-2 expression.Since CS-6 targets IKKs to suppress COX-2 expression and CB is a COX-2-selective inhibitor, we next detected the combined effect of CS-6 and CB on IKK signaling. The A549 cells were pre-treated with CB (50 uM) for 8 h and then treated with CS-6 (50 nM) for 48 h. As shown in Figure 6D, the combined treatment of CB and CS-6 effectively decreased the level of p-IKKβ without affecting the total IKKβ expression. This effect was similar to CS-6. These results suggest that CS-6 might be an inhibitor of COX-2 like CB.

### CS-6 inhibited the growth of lung cancer xenografts in nude mice

**Figure 7:**
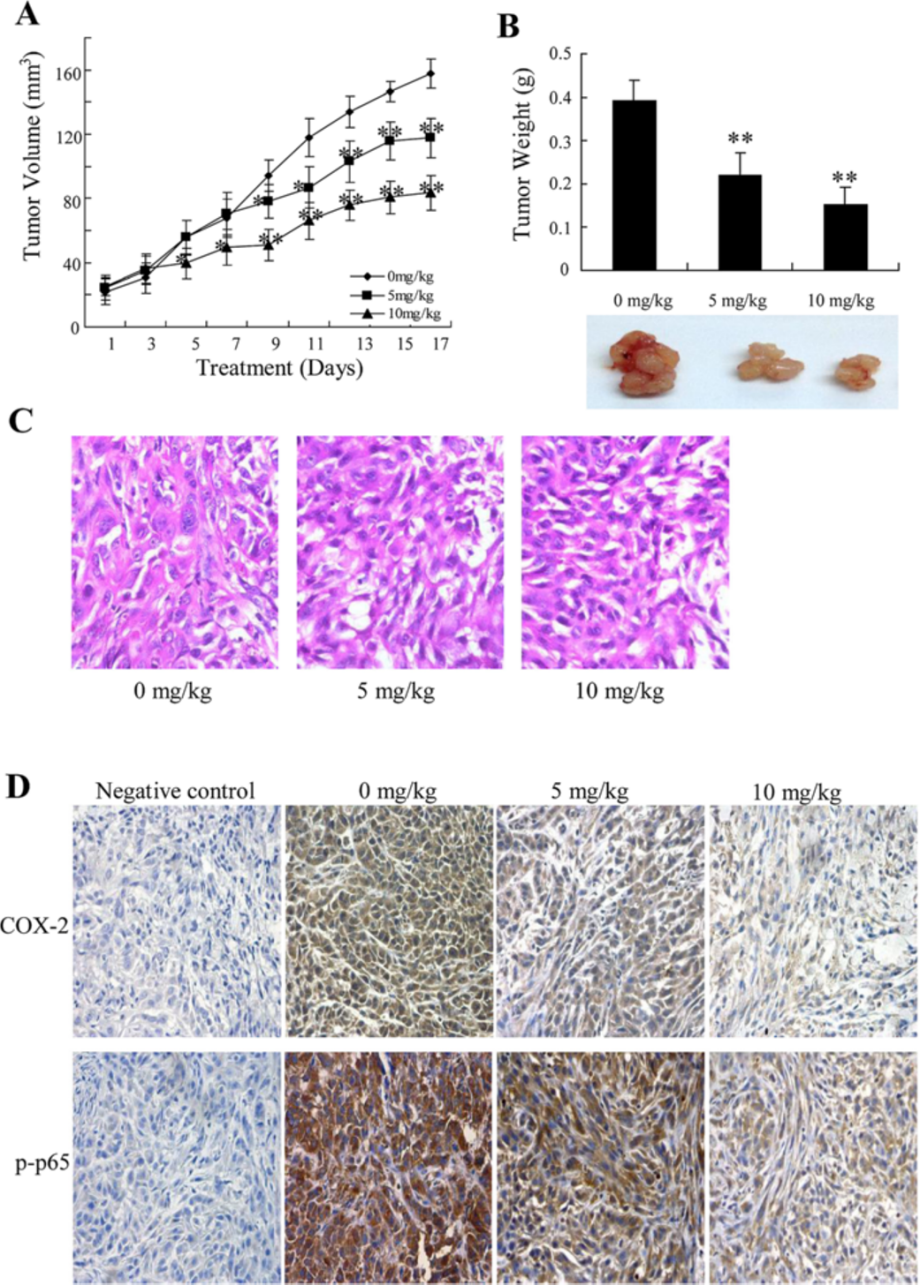
**CS-6 inhibited tumor growth in lung cancer mouse models.** An orthotopic mouse model of human NSCLC A549 was used to evaluate the effect of CS-6. Tumor volumes **(A)** and total weights **(B)** were measured. **(C)** H&E staining. **(D)**. Immunohistochemical analysis of COX-2 and p-p65 protein expression in tumor samples. Neutral formalin fixed tumor samples were prepared from animals and analyzed by immunohistochemical staining with rabbit anti-rabbit second antibody using the Vectastain Elite ABC kit, and examined under a microscope.

Based on the results of *in vitro* studies, we further explored the potential of CS-6 as a novel molecular therapeutic agent for tumor growth in mice with human lung cancer xenografts. Mice bearing subcutaneous tumors were treated with therapy 14 d after tumor cell injection. Mice were divided into three treatment groups. After administration of CS-6 at 5 and 20 mg/kg/day in the mice with A549-xenografts for 17 days, both the tumor volume (Figure 7A) and the tumor weights (Figure 7B) in the treated mice decreased significantly when compared with those in the control group. No obvious toxic effects in mice treated by CS-6 were detected. In addition, H&E staining also showed that the untreated tumor cells were irregular and had abundant cytoplasm, large and deformity nuclei and high nucleocytoplasmic ratio. The nuclear pleomorphism and nucleolus were prominent. It could be also seen amphinucleolus and mitotic (Figure 7C). However, in treatment group tumor cells, it was rarely seen amphinucleolus and mitotic, and the nucleolus was smaller and more regular (Figure 7C). Moreover, the immunohistochemical staining assay was used to determine the expression of COX-2 and p-p65. The expression levels of COX-2 and p-p65 were significantly decreased with CS-6 treatment *in vivo*, as compared with the vehicle group (Figure 7D). These results supported that CS-6 could inhibit the xenografted human lung cancer cell’s growth without the remarkable adverse effects.

## Discussion

Toad Venom, a traditional Chinese medicine (TCM), is an anti-inflammatory drug used in small doses for the treatment of various types of inflammation in China [32, 33]. Bufadienolides, such as bufalin and cinobufagin as its major active ingredients, exhibited the significant antitumor activities, including inhibition of cell proliferation, induction of apoptosis, inhibition of cancer angiogenesis, and regulation of the immune response [32]. Meantime, Toad Venom extracts or bufadienolides also exhibited the significant anti-inflammatory action by inhibiting the proliferations of human T-lymphocytes [33]. However, there is little known about the biological effects of other toad venom ingredients *in vivo* and *in vitro*. Gamabufotalin (CS-6) is a major bufadienolide with better drug ability, but only few researches have studied on its biological activities.

In the present study, we found that gamabufotalin (CS-6) could effectively inhibit NSCLC cells growth and enhance apoptosis induction dose-dependently with IC_50_ of only 50 nM, while CS-6 nearly had no adverse effect on human normal lung tissue cells at the dosage of 10 μM. Furthermore, we showed that the effects of CS-6 on lung cancer cells growth and apoptosis were mediated through inhibiting NF-κB/COX-2 signaling pathway and activating the cytochrome c/caspase-dependent apoptotic pathway. CS-6 inhibited COX-2 expression through suppressing the phosphorylation of IKKβ by targeting the ATP-binding site, thereby inhibiting translocation of the NF-κB p65/p50 proteins from cell cytoplasm to nucleus and abrogating NF-κB binding and p300 recruitment on COX-2 promoter. Here, to the best of our knowledge, it might be the first time to report the treatment of CS-6 on COX-2 expression and to demonstrate the underlying mechanisms both *in vitro* and *in vivo.*

In our study, considering that A549 cell line overexpresses COX-2 and has a higher ability to form xenograft in nude mice, we performed almost *in vitro* experiments in A549 cells to study the molecular mechanism of CS-6 suppressing COX-2 expression.

One of the pivotal roles in the inflammatory processes is cyclooxygenase-2 (COX-2), an inducible enzyme, which can be rapidly induced by inflammatory mediators, cytokines, growth factors and tumour promoters [34–36]. Previous studies have shown that COX-2 overexpression has a significantly central role to in cancer development by promoting cell proliferation, decreasing apoptosis rate, and increasing invasive and metastatic potential of the primary tumor [37–39]. To clarify the mechamism of CS-6 from Chansu used as an anti-cancer agent, we investigated whether COX-2 plays an important role in CS-6 bioactive function, and found CS-6 could inhibit COX-2 expression, along with inhibiting NSCLC viability, migration and colony formation.

The transcription factor NF-κB has been shown to be involved in COX-2 expression in various cell types [40]. Transcriptional coactivator p300 could increase the transcriptional activity of the NF-κB complex through modification of chromatin structure and the direct acetylation of p65 and p50 [41]. These evidences suggested that the activation of NF-κB complex p300 played an important role in bridging the multiple DNA-bound transactivators with transcription factors to initiate COX-2 transcription. In our study, we confirmed the nuclear localization and interaction of NF-κB and p300 in lung cancer cells, and found that CS-6 inhibited NF-κB translocation from cytosol to nuclear and its binding to COX-2 promoter, abrogating COX-2 transcriptional activation, thereby reduce COX-2 expression. In our study, we found that CS-6 inhibited COX-2 expression and induced apoptosis; however, no direct correlation between them was observed.

NF-κB is kept in an inactive state in the cytoplasm by interacting with members of the IκB family of proteins which mask the nuclear translocation signal of NF-κB [42]. Upon stimulation, IκB proteins become phosphorylated at Ser32 and Ser36 residues by the inhibitor of κB (IKK) kinase complex, ensuing degradation. Therefore, IKK is essential to NF-κB activation. Next, we studied whether CS-6 could affect IKK activity. Our present study strongly indicated that CS-6 could inhibit serine phosphorylation of IKKβ in a dose-dependent manner. Moreover, computational docking implied that CS-6 occupied the deep hydrophobic pocket in the ATP-binding site of IKKβ. In this modeling analysis, CS-6 located well in the ATP binding site and interacted with the hinge region backbone residue Cys99, and also makes hydrogen bond interaction with Glu149, same as K-252A, which may be another reason for higher inhibition activity [43, 44]. Our results suggested that CS-6 might block the nucleotide recognition domain binding with ATP, as a reversible inhibitor. This is just consistent with our experimental results. Hydrophobic interactions should be emphasized because the ATP binding pocket is consisted of a narrow and hydrophobic region. These data mentioned above suggested that CS-6 may attenuate the transcriptional activity of NF-κB, at least in part, by abrogating the activity of IKKβ. IKKα and IKKβ are the two catalytic subunits of IKK, and have a high degree of sequence homology and share similar structural domains. However, previous studies have clearly demonstrated that IKKβ subunits of IKK complex are required for NF-κB activation by all known pro-inflammatory stimuli including lipopolysaccharide (LPS), TNFα and IL-1β [45, 46]. Thus, we mainly focus on the effect of CS-6 on IKKβ in the present study.

In conclusion, we found that CS-6 suppresses COX-2 expression in lung cancer, both *in vivo* and *in vitro*. Mechanistic investigation reveals that CS-6 may target IKKβ signaling cascades. These findings provide strong evidences for potential of CS-6 to be a novel anti-cancer agent in NSCLC treatment.
