# Correction: Gamabufotalin, a bufadienolide compound from toad venom, suppresses COX-2 expression through targeting IKKβ/NF-κB signaling pathway in lung cancer cells

**DOI:** 10.1186/s12943-023-01852-5

**Published:** 2023-09-02

**Authors:** Zhenlong Yu, Wei Guo, Xiaochi Ma, Baojing Zhang, Peipei Dong, Lin Huang, Xiuli Wang, Chao Wang, Xiaokui Huo, Wendan Yu, Canhui Yi, Yao Xiao, Wenjing Yang, Yu Qin, Yuhui Yuan, Songshu Meng, Quentin Liu, Wuguo Deng

**Affiliations:** 1 Institute of Cancer Stem Cell, College of Pharmacy, Dalian Medical University, Lvshun South Road No 9, 116044 Dalian, China; 2 State Key Laboratory of Oncology in South China, Collaborative Innovation Canter of Cancer Medicine, Sun Yat-sen University Cancer Center, Guangzhou, China

**Correction:** ***Mol Cancer*** **13, 203 (2014)**

10.1186/1476-4598-13-203

Unfortunately, the original version of this article [1] contained some errors. The words “binding” has been spelt incorrectly in the fifth title of result section and legend of Fig. 5. And the image of GAPDH (Fig. 6D) has been mistakenly uploaded. The mistakes did not affect any correctness of our results or discussion. The corrected sentence Fig. 6 was provided here. We regret any inconvenience that caused.

“CS-6 inhibited NF-κB and p300 translocation and binding to COX-2 promoter”. “Figure 5 CS-6 inhibited NF-κB and p300 translocation and their binding to COX-2 promoter”.

**Fig. 6:**
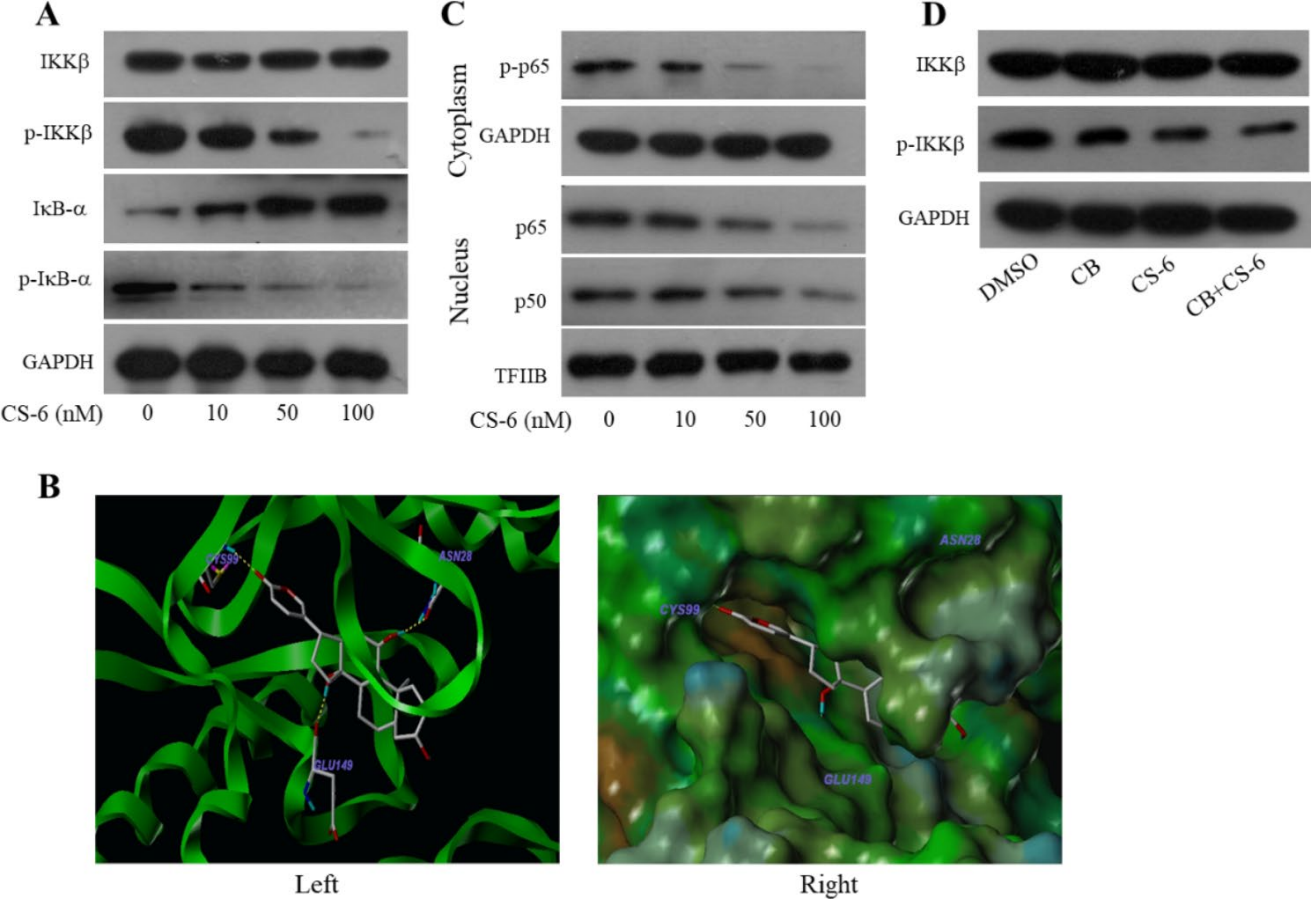
**CS-6 inhibited the phosphorylation and activation of IKKβ.(D)** A549 cells were treated with CS-6 (50 nM) after pretreatment with CB (50 nM). The IKKβ, and p-IKKβ proteins were analyzed by Western blotting.
